# Paranormal experiences, sensory-processing sensitivity, and the priming of pareidolia

**DOI:** 10.1371/journal.pone.0274595

**Published:** 2022-09-14

**Authors:** Jess M. Williams, Mark Blagrove

**Affiliations:** Department of Psychology, Swansea University, Swansea, Wales, United Kingdom; University of Granada: Universidad de Granada, SPAIN

## Abstract

This investigation tested the effect of priming on pareidolia (the hearing of illusory words in ambiguous stimuli). Participants (41 women, 20 men, mean age 29.95 years) were assigned to primed (n = 30) or unprimed (n = 31) groups: the former were told the study was of ‘purported ghosts voices’, the latter ‘voices in noisy environments.’ Participants were assessed for perception of human voices within recordings of purported electronic voice phenomena (EVP), degraded human speech, normal human speech, and white noise. The primed group had significantly higher perception of voices within EVPs than in degraded speech, this difference was not found for unprimed participants. In contrast to the previous use of this design, the primed group did not have higher perception of voices in EVPs and degraded speech than did the unprimed group. The Aesthetic Sensitivity dimension of the Highly Sensitive Person Scale (HSPS) was associated with detection of degraded stimuli, but not with accuracy of stimulus identification. HSPS score was related to lifetime reporting of anomalous and paranormal experiences. This study partially replicates a paranormal priming effect and shows relationships between HSPS and detection of ambiguous stimuli and anomalous and paranormal experiences.

## Introduction

Although paranormal phenomena defy what is deemed physically possible [1], there remains frequent reporting of such experiences within the general population [2, 3]. Attempts have been made to explore reasons for this reporting through studies on human perception and personality. The present study addresses perceptual and personality correlates of one type of paranormal phenomenon, that of electronic voice phenomena (EVP), defined as apparent ghost voices attempting to communicate through typical audio recording equipment. Although these ghost voices may be nothing more than random audio signals within white noise, interpreted by the perceiver as containing spoken content [4], they are commonly accepted as evidence of the paranormal, and the vulnerability to perceive these as paranormal recordings has been previously addressed by the literature.

The assumption behind the scientific approach to these perceptual illusions is that susceptibility to the illusions is a result of evolutionary pressures for our ancestors in detecting threats and subtle social cues [5]. In particular, EVP is an example of an illusion that results in the perception of meaningful patterns in meaningless stimuli, referred to as pareidolia, occurring in both visual and auditory forms, such as seeing faces in clouds or hearing phantom words in white noise. Acting as a mechanism for specific paranormal events, a susceptibility to pareidolia may be associated with the holding of paranormal beliefs. For instance, Riekki et al [6] demonstrate that paranormal believers are more likely to perceive faces in images regardless of whether face-like cues are present. Believers also report hearing human voices in ambiguity, whereas disbelievers report more sound-like signals such as sirens and swooshing [7]. This may be explained by certain perceptual experiences being consistent with pre-existing paranormal beliefs, leading to paranormal interpretations of such experiences [8, 9], closely related to the effects of suggestibility.

Prior expectations and suggestion are also proposed to impact on perceptual experiences. Halligan and Oakley highlight the effects of priming, such that prior knowledge or belief can influence individuals’ thoughts, behaviour, and emotions, and exposure to paranormal suggestion can result in the reporting of paranormal phenomena [10]. As an example, Wiseman and colleagues [11] created a series of fake seances, and suggested to participants that an object was moving, although no movement occurred. When asked later in the experiment, 31% of participants falsely recalled that the object had moved, revealing that the mere suggestion of paranormal forces created false memories and paranormal experiences [12]. Interestingly, this false reporting was significantly associated with paranormal belief. Similarly, Drinkwater et al [13] found that 36% of their participants reported hearing voices in random noise after watching videos informing them about EVP, demonstrating that the prior education and expectation of EVP introduces misperceptions of such within pure noise.

Nees and Phillips [14] aimed to test whether a simple instructional prime could lead to a perceptual shift, resulting in a higher reporting of voices within ambiguity. A total of twenty-seven psychology students were recruited and assigned to either a primed or unprimed group. The primed group were told it was a paranormal study, the unprimed group were left naïve to the true nature of the experiment. All participants listened to a series of audio recordings, containing examples of EVP, degraded human speech, normal human speech, and pure white noise. The results revealed that primed participants detected more voices within ambiguous speech conditions than did unprimed participants. However, there was no agreement across participants of the spoken content of the EVP stimuli. Baker suggests that where sensory information is limited, top-down processes attempt to fill these gaps, resulting in misperception, and potential reporting of paranormal phenomena [15, 16]. A limitation of this study, however, was that the small and sceptical sample resulted in the inability to compare believers and non-believers on their perceptions and susceptibility to priming.

Additionally, a temperament trait that has been associated with both perception and paranormal belief/experiences is sensory-processing sensitivity (SPS), characterised by an increased sensitivity to the environment and an enhanced, deeper processing of stimuli, sensory information, and emotions. To describe individuals who experience high SPS, Aron [17] devised the term Highly Sensitive Person (HSP), and the Highly Sensitive Person Scale (HSPS) was developed to measure SPS by Aron and Aron [18]. This questionnaire measures aspects of behaviour and perceptual experiences associated with being a HSP, such as being sensitive to caffeine, becoming overwhelmed by large levels of sensory input, and being perceived as sensitive or shy. Interestingly, SPS has been viewed as a negative trait within some literature, posing detriments to the daily functioning of HSPs. For instance, in therapeutic environments, SPS is confused for other disorders such as obsessive-compulsive disorder, anxiety, and depression [19]. It has also been associated with negative personality traits such as neuroticism [20, 21], as well as mental health vulnerabilities [22], poor stress management [23] and nightmare distress [24, 25].

Consistent with the differential susceptibility framework, HSPs experience intense positive reactions to positive stimuli, and negative reactions to negative stimuli. Thus, it can be described as a *for better or for worse trait*. The previous literature has mainly focused on the *for worse*, associating SPS with negative emotionality and mental disorders, leading to the inflated reporting of SPS resulting in negative reactivity to the environment. However, given the correct environment, HSPs are able to thrive, and function adequately and effectively, with Aron [19] describing the benefits of having greater empathy and being more creative.

An additional characteristic of this trait is a potential perceptual advantage, due to a greater depth of processing and awareness of external subtleties [26]. Jagiellowicz et al [27] tested participants’ responses and brain activation to subtle changes of scenes in a change detection task. Participants high in SPS took longer to respond to minor (versus major) scene changes, thought to be a result of closer attention to the subtle details of the scene. There also seemed to be higher levels of activation within the visual and attentional areas of the brain during such changes, once again highlighting deeper processing of the images. In addition, Gerstenberg [28] found that high SPS was associated with faster reaction times and fewer error rates on a visual detection task, suggesting an increased processing of visual information, but also the consequence of increased stress levels.

Although Aron and Aron [18] intended for the HSPS to be a unidimensional measure of SPS, there is conflicting research suggesting its multi-dimensionality. Smolewska, McCabe, and Woody [29] proposed there were three subscales of the HSPS. The first, Ease of Excitation (EOE), suggests a vulnerability to becoming mentally overwhelmed by external stimuli. Secondly, Aesthetic Sensitivity (AES) describes an “aesthetic awareness”, and thirdly, Low Sensory Threshold (LST) refers to becoming unpleasantly aroused by external stimuli. Evans and Rothbart define AES as Orienting Sensitivity, relating to automatic attentional processing [30] and interestingly, this subscale seems to represent a different type of sensitivity to EOE and LST. For instance, Elst et al [31] found that EOE and LST amplified the associations between job demands and emotional exhaustion in the workplace, and whereas these were considered personal vulnerability factors, AES acted as a personal resource, finding associations with both increased helping behaviour and decreased emotional exhaustion. AES also positively correlates with openness to experience and holds no associations with negative personality traits, such as neuroticism [29].

This suggests that AES represents a positive element of high SPS, in that it results in deeper, aesthetic processing of subtleties [32], whereas EOE and LST seem to result in overstimulation in response to the environment and sensory experiences [33], signifying negative effects [30]. Thus, we will test whether the perceptual advantage of SPS, the ability to detect and process subtleties within the environment, is associated with the positive AES element of SPS and/or the negative facets of SPS (EOE/LST). Although the previous research claims that AES is associated with these automatic sensory processes, there is no behavioural evidence that has tested this.

Furthermore, many studies have addressed the relationship between reporting of paranormal phenomena and other types of sensitivity, such as thin boundariness [34–36] and heightened environmental sensitivity [37–39]. There is limited research, however, which evidences the association between the paranormal and SPS, although Irwin and colleagues [40] and Williams et al [41] (using the HSPS and the Survey of Anomalous Experiences [42]) found that those high in SPS report a greater number of anomalous experiences with paranormal attributions. Further research is required to support this heightened reporting of paranormal experiences in HSPs.

With these apparent relationships between SPS, paranormal experience and perception, a paradox emerges in that SPS and paranormal experiences are differentially related to perception although related to each other. For example, Williams et al [41] demonstrated that higher reporting of paranormal experiences was positively associated with illusory perception (pareidolia), that is the misperception of words within random noise [43, 44]. Paranormal experiences were also related to SPS scores. However, SPS was not associated with misperception, but instead to the accurate detection of degraded words. Therefore, this suggests that both SPS and pareidolia are predictors of paranormal experiences, but are independent of one another, possibly explaining the observed differences in relationships.

### Aims and hypotheses

The methods of Nees and Phillips [14] were used for the current investigation due to the appropriateness of the stimuli in testing perceptual accuracy and pareidolia. Participants were split into two groups; one group received a simple instructional prime that informed them of a paranormal nature to the study. The other group were not primed and instead told it was a study about noisy recordings. The participants listened to the same four conditions of speech recordings (34 recordings each of human speech, degraded human speech, white noise, and EVP) and were instructed to state whether or not they heard a voice after each presentation. Once the task was completed, they filled in the HSPS and Survey of Anomalous Experiences (SAE) to measure SPS and paranormal experiences (respectively). The SAE provides participants a list of potential paranormal experiences which they could have encountered, and they were to state whether they have had the experience and would attribute it as paranormal or give it a natural explanation, or simply that they have not had the experience.

The aim of this study was to test the associations between paranormal experiences, SPS, priming, and perception (including accurate perception and misperception). Firstly, although the previous literature has demonstrated believers’ susceptibility to suggestion effects (e.g., French [12]), there is currently a lack of understanding in terms of the relationships between paranormal and perceptual experiences. In particular, the research suggests that believers and experiencers (although limited for the latter) show an enhanced susceptibility to perceptual illusions, such as pareidolia, potentially providing one mechanism for the reporting of paranormal encounters whereby ambiguous stimuli in the environment are interpreted as being paranormal [6, 41]. It is therefore expected that those demonstrating an increased reporting of paranormal phenomena will be more likely to detect voices within the ambiguous and eerier-sounding EVP recordings, which can be defined as pareidolia [4]. Also, due to apparent heightened proneness to priming in believers of the paranormal [11], it was also hypothesised that participants with higher numbers of paranormal experiences will hear more ambiguous voices within EVP when primed to expect paranormal recordings. There were no directional hypotheses between the relationships with the other conditions of speech, although these were assessed.

Secondly, due to the sparseness of literature focusing specifically on SPS and paranormal experiences, these associations were examined. Thirdly, this study also tested the perceptual advantage of high SPS. Williams et al [41] highlight that high SPS is associated with the detection of subtle, degraded stimuli, and not pareidolia, supporting a lower filtering of incoming information, assisting in processing true signals in the environment, and is a potential evolutionary advantage. Thus, it is hypothesised that SPS (and possibly the facets of SPS, specifically AES) will correlate with accurate detection of true, degraded stimuli and not with pareidolia. That is, the proportion of voices detected within the degraded human speech recordings, which contain real voices that have been audibly degraded to create difficulty with accurate speech perception.

Finally, the study aimed to replicate the results of Nees and Phillips [14]. The authors found that participants told of the paranormal nature of the study were more susceptible to reporting voices in all stimulus types, although in general, the perception of voices were similar between the EVP and degraded human speech recordings (two types of ambiguous stimuli). They also demonstrated that participants were unable to accurately identify the spoken content of the EVP messages, as well as the lack of perceptual consistency amongst participants of these recordings. In-line with this research, it was firstly expected that paranormal primed participants would be more susceptible to hearing voices in all stimulus types, especially the ambiguous stimulus conditions of EVP and degraded human speech. It was also hypothesised, from the findings of Nees and Phillips [14], that there will be no difference between the perceptions of voices within the degraded speech and EVP conditions, due to the ambiguity of these stimuli. However, the degraded speech stimuli contain true signals, and thus it is possible that this stimulus condition will demonstrate a higher percentage of detected voices compared with the pareidolic stimuli of EVP, this was therefore explored. Also, due to difficulty with non-contextual speech perception, participants would also be unable to reliably identify purported messages in EVP stimuli (due to the absence of human speech) as well as within the degraded speech (due to sufficient degradation creating difficulty with speech perception). Furthermore, it was expected that there would be a lack of agreement between participants’ perceptions of the EVP spoken words and the perceptions of the original paranormal researchers. The current study intended to extend these findings through the addition of the SAE and HSPS with a larger sample, which is an advantage as the authors were limited due to their sample’s overall scepticism of the paranormal. Additionally, Signal Detection Theory (SDT) was applied to support relationships with perceptual experiences, providing the ability to differentiate between true perception and response bias [45].

## Materials and methods

### Participants

62 participants were recruited through social media and the university psychology credit system. Participants were awarded either university credits or a raffle ticket to win a prize. One participant did not complete the computer task due to program failure, and their data were excluded. There was a total sample size of *N* = 61, with 20 men and 41 women, ages 18 to 54 years (Mean = 26.95 years, SD = 9.86), and 90.2% were White. 13.1% of participants were educated to high school level (or below), 46.5% had completed college or sixth form (A-Level), 31.3% achieved Bachelor’s Level (undergraduate), and 8.2% achieved Master’s Level qualifications. Full written consent was provided by participants. The study was granted ethical approval from Swansea University’s College of Human and Health Sciences Research Ethics Committee.

#### Sample size, power calculation and sensitivity analyses

Nees and Phillips [14] found two significant main effects (stimulus type: η_p_^2^ = .92; group: η_p_^2^ = .15) as well as a significant interaction (η_p_^2^ = .15). The minimum effect size of η_p_^2^ = .15 (Cohen’s F = .42) inputted into G*Power software [46] revealed a necessary sample of *N* = 50 to achieve adequate power (95%, alpha = .05) for the between-subjects main effect and interaction. However, due to the potentially inflated effect sizes found by the original authors, this might have been an underestimation of the required sample. 61 participants were thus collected, and although the authors aimed to recruit more, the restrictions posed during the COVID-19 pandemic and time restraints did not allow for this. A sensitivity analysis found that a sample of *N* = 61 (95% power, alpha = .05) was sensitive enough to detect an effect size of Cohen’s F = .19 (η_p_^2^ = .003) for the within-subjects main effect and within-between interaction, and an effect size of Cohen’s F = .37 (η_p_^2^ = .12) for the between-subjects main effect.

### Materials

#### Stimuli

The stimuli were those used in Nees and Phillips [14]. There was a total of 136 stimuli arranged in four conditions (34 stimuli per condition). Each stimulus lasted approximately 600 milliseconds, including a 5-millisecond onset given by the original researchers. The four conditions were EVP, human speech, degraded speech, and artificially produced white noise. The EVP recordings were examples of purported ghost voices captured during real paranormal investigations and can be defined as pareidolia [4]. Human speech recordings included clearly spoken words or phrases, such as “*Anything*”, “*Is there anybody*”, and “*Talk to us*”. The degraded speech stimuli were the same recordings used in the speech condition, degraded within white noise, with the signal to noise ratio creating challenges for speech perception. The speech and white noise stimuli acted as control conditions for the EVP and degraded speech conditions. All audio files (except for the artificial white noise condition, created using Audacity) were extracted from a series of videos titled *Ghost Adventures—Guess that EVP* on the Travel Channel Website (see Nees & Phillips [14] for details of stimulus production).

#### Questionnaires

The Survey of Anomalous Experiences (SAE) [42] was used to measure the extent to which participants report having previous paranormal experiences (Cronbach’s alpha = .83). This questionnaire provides 20 anomalous experiences and participants respond according to whether they have had the experience and would attribute it as paranormal, if they have had the experience but believe there was an alternative explanation, or that they have never had the experience. Questions 14 and 19 were reworded to create a broader anomalous experience which could apply to more individuals, and the first response option for all items was reworded to “*I think it was…*” instead of “*It must have…*”. For instance, “*I have become aware of a scent in a room*, *yet there was nothing there that could have that smell*”, gives the following response options: “*Yes*, *and I think it was an instance of an apparition or ESP*.” (Paranormal Response), “*Yes*, *but it was probably just an illusion or physiological anomaly*.” (Anomalous Response), or “*No*” (No Response). The total number of responses per option per participant was calculated, resulting in three variables of SAE No Responses, Paranormal Responses, and Anomalous Responses.

To measure SPS, the Highly Sensitive Person Scale [18] (HSPS; Cronbach’s alpha = .85) was used, which asks about the sensory experiences of the individual. Participants responded to 27 questions with reference to a 7-point Likert scale (1 = Not at all; 7 = Extremely). Questions include, “*Are you easily overwhelmed by strong sensory input*?” and “*Do you startle easily*?”. The responses to each question were added together to give a total score, and a mean score was calculated, with a higher score representing higher SPS. The mean scores for each of the subscales were also calculated according to Smolewska et al.’s three subscales of AES (7 items; a = .68), EOE (12 items; a = .75), and LST (6 items; a = .68) [29].

### Procedure

The current design was a replication of the procedure used by Nees and Phillips [14], with the addition of the SAE and HSPS. Firstly, participants were asked to read an information sheet that contained all the relevant information regarding the study. The title given to all was “*Voice Detection in Sounds*”, so that participants in the unprimed group remained naïve to the paranormal aspect of the investigation. All provided informed written consent. Participants sat in front of a MacBook Air (2017) and placed a pair of Sony MDR-ZX310APB headphones over their ears.

#### Priming manipulation and computer task

The computer program randomly assigned participants to one of two groups, either the paranormal primed group (n = 30) or the unprimed group (n = 31). The only difference between these two groups was the first line of the instructions. The primed group read the following, *“This is an experimental study on the identification of electronic voice phenomenon–purported voices of ghosts in recordings from paranormal research*.*”*, whereas the unprimed group were told, “*This is an experimental study of the identification of voices in noisy environments*.” The remainder of the instructions were the same for both groups, that they would hear a variety of sounds in the headphones, and after hearing each sound they should respond ‘Yes’ or ‘No’, depending on whether they heard a voice in the recording. To move on from the instructions, participants pressed the spacebar, and a proceed screen was displayed, asking participants to, *“Please press the spacebar to begin a trial*.*”*. This appeared before each of the 136 trials (stimuli) to ensure participants were prepared for and not surprised by the sounds. After pressing, a blank screen appeared, and the stimulus played after a 1000ms onset. Once the recording had finished, the program moved to the question screen automatically, asking the participant if they heard a voice in the sound. If they responded yes, they were immediately directed to an additional question screen, which asked participants to type in a box a guess as to what they thought the voice had said, and to not give responses such as “*I don’t know*” or “*unsure*”. If they responded no to hearing a voice, they immediately moved to the next trial proceed screen. An unlimited amount of time was given to answer, and the stimuli were presented in a different random order for each participant. The percentage of yes responses per condition was calculated by dividing the total number of yes responses in that condition by the number of trials (34 trials), as a measure of the propensity to perceive human voices within the recordings. In addition, participants’ guesses as to the spoken content of the recordings were extracted and used to test for spoken content agreement.

#### Questionnaires

Once the computer task was completed, participants were asked to fill in electronic versions of the SAE and HSPS on Qualtrics (Qualtrics, Provo, UT) using an Apple iPad Air (2018). Data were also collected regarding participants’ age, gender, education level, and ethnicity. Participants were fully debriefed at the end of the study, the purpose of the investigation was explained, and their group assignment was revealed. The final question on the questionnaire asked participants if they had guessed the true nature of the experiment, i.e., that there was a paranormal nature and/or that they had been randomly assigned to one of two groups. This was specifically important to ensure the unprimed group remained naïve to this throughout, so the priming manipulation was not diminished, and they remained unaware of the paranormal aspect of the investigation. One example of how they could have been aware of this during the experiment is if the participant had recognised the voices of the paranormal investigators from the television show “Ghost Adventures”. Another reason was to control for diffusion of the instructional manipulation that occurred, e.g., amongst class peers, a previous limitation of the original study [14]. One participant did not give a response as there was an error with the questionnaire software.

### Statistical analyses

All analyses were conducted using IBM SPSS Statistics for Macintosh, Version 26.0 and Version 28.0 (IBM Corp, Armonk, N.Y, USA), graphs were created using Python Programming Language (version 3.8.8) and Microsoft Excel (version 16.57). The variables of interest measured perception (percentage of yes responses across the four stimulus conditions), paranormal experiences (SAE Responses), and SPS (HSPS Score and scores on each subscale). Bivariate correlations were conducted between these to test for associations. A multiple regression was also used to test the distinct relationships of SPS and paranormal experiences with perception, in that SPS and pareidolia were expected to be independent predictors of the reporting of paranormal experiences.

#### Signal detection analysis

To investigate and support associations between perception and SPS, Signal Detection Theory (SDT) measures were used to distinguish true perceptual sensitivity from a tendency to respond with bias. Responding “Yes” in the degraded speech condition were considered “Hits” and responding “Yes” in the noise condition were considered “False Alarms”. Scores of zero were replaced with $\frac{0.5}{34}$ = 0.014 and d’ (sensitivity) and β (response bias) were calculated per participant, according to the method detailed by Stanislaw and Todorov [47].

The analyses of Nees & Phillips [14] were replicated. A 2 (priming group) x 4 (stimulus condition) mixed analysis of variance (ANOVA) was conducted, with the proportion of yes responses as the dependent variable. A 2 (priming group) x 2 (stimulus condition) ANOVA was also conducted using only the detection of voices within the EVP and degraded speech stimulus conditions, due to the non-normality of the speech and noise conditions (Nees & Phillips [14]; footnote 2, p29). Paranormal Responses and SAE No Responses were included as covariates to control for participants’ existing susceptibility to experience paranormal phenomenon as well as suggestibility. The current study involves priming, stimulus detection, and paranormal experiences, and thus all variables are present within the ANOVAs.

It is important to note the potential confound that is introduced in that the recordings of human speech were the basis also of the stimuli in the degraded human speech condition. Thus, it is possible that an advantage due to priming is present for degraded human speech stimuli that are presented after the speech equivalent. The task order effect was analysed for n = 40 participants (specific order of presentation data were unavailable at time of analysis for the remaining participants), for whom there was a mean of 17.55 (SD = 2.54) degraded stimuli presented before the speech equivalent and 16.45 (SD = 2.54) presented after, there was no significant difference between these, *t*(40) = 1.37, *p* = .18. This did result in a priming effect, the mean percentage of voices detected was 50.24 (SD = 21.70) for degraded words presented after the non-degraded words, and 44.23 (SD = 22.72) when presented before, *t*(40) = -2.151, *p* = .038. As the order of presentation of spoken word and degraded word stimuli was balanced by randomisation, order of presentation was not included as a factor in the ANOVA and regression analyses.

Qualitative data (participants’ guesses) were collected and analysed for any agreement amongst participants and a one-way ANOVA was conducted to test the difference between the maximum agreement in each stimulus condition (34 trials each), with each trial as a case. Participants’ agreement with the original interpretations of the EVP stimuli (according to the subtitled videos in which the EVP originated) were also compared to test for spoken content agreement; the number of times in which participants agreed with these interpretations was calculated.

## Results

**Fig 1:**
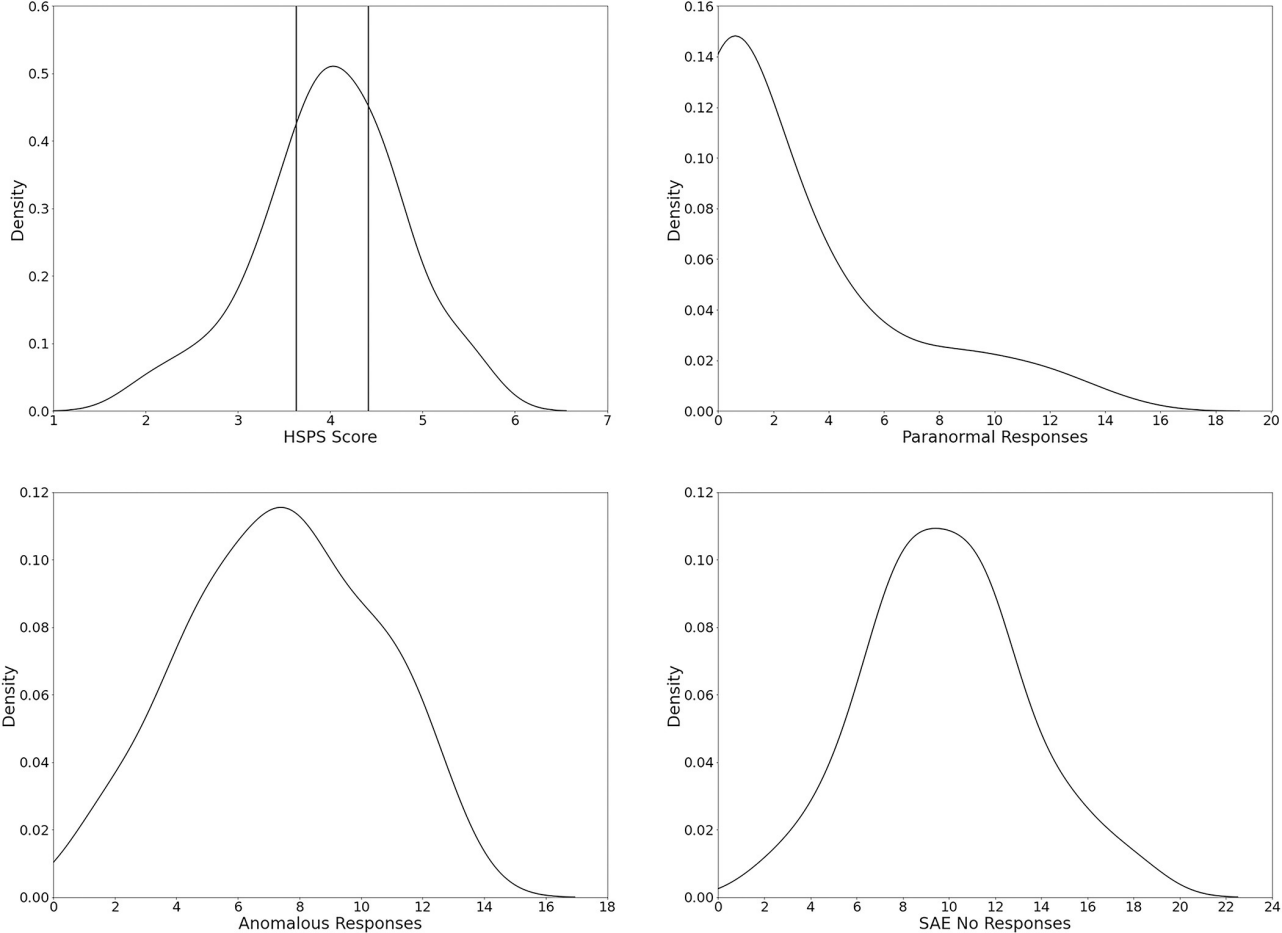
Density distribution of mean HSPS scores, total Paranormal Responses, Anomalous Responses, and SAE No Responses. For HSPS score, lines represent cut-off points for SPS groups as according to Lionetti et al [48]. The first line is the cut-off between the low SPS group (29.5% of the sample) and medium group (HSPS score = 3.63) and the second line for medium and high SPS group (31.1% of the sample) (HSPS score = 4.41). Density distributions for the subscales of the HSPS can be found in the S1 Fig.

Density distributions of HSPS score and the SAE responses are displayed in Fig 1. The assumption of normality was violated for the percentage of yes responses in the speech and noise conditions, SAE Paranormal Responses, as well as for EOE (Shapiro-Wilk, *p* < .05). The mean HSPS score was 3.99 (SD = 0.78), suggesting the sample was less sensitive than in Aron and Aron [18] but is comparable to other studies on SPS (e.g., [41, 48, 49]). Independent t-tests revealed no significant differences between males and females for any of the variables (all *p*s > .05) and age was significantly correlated with the number of SAE No Responses (*r*(61) = .267, *p* = .04) and Anomalous Responses (*r*(61) = -.343, *p* = .007), as well as with HSPS Score (*r*(61) = -.39, *p* = .002).

57% of participants reported having at least one prior paranormal experience. The paranormal experience with the most Paranormal Responses was item 5, which states “*On at least one occasion*, *I’ve had the impression of a figure nearby*, *yet nobody could possibly have been there*”. 20 participants (32.8%) believed they had seen an apparition or ghost. On the other hand, the least common paranormal occurrence was item 16 “*In a life-threatening situation I have had the impression that my disembodied ’self’ was moving along a tunnel toward a light*.” Only one individual had experienced this and attributed it as paranormal, the remaining 60 participants (98.4%) responded “No”.

All correlations statistics are displayed in Table 1. Relationships between the SAE responses and voice detection were non-significant (*p* > .05) across all stimulus conditions, and this remained the case when correlations were conducted separately for the primed and unprimed groups (S1 and S2 Tables). HSPS score was positively associated with the number of Paranormal Responses, *r*_*s*_(61) = .298, *p* = .02, as well as negatively related to the number of SAE No Responses, *r*(61) = -.301, *p* = .02. This suggests that higher SPS is associated with an increased reporting of paranormal experiences. The subscale AES also had significant associations with SAE No Responses (*r*(61) = -.44, *p* < .001) and Paranormal Responses (*r*_*s*_(61) = .28, *p* = .03). Furthermore, the only significant correlation found for SPS and perception was that between AES and the percentage of degraded words detected, *r*(61) = .30, *p* = .02, a higher AES score was related to higher perception of words within this stimulus condition.

**Table 1:** Correlations between the perception, paranormal experiences, and sensory-processing sensitivity.

	SAE No Responses	Anomalous Responses	Paranormal Responses	HSPS Score	EOE	LST	AES
***Percentage of Words Detected*:**							
Degraded Human Speech	-.214	.055	.117^a^	.116	-.122^a^	.118	.300^a^
Human Speech	-.009^a^	.140^a^	-.021^a^	.062^a^	.236^a^	.131^a^	-.137^a^
EVP	-.123	.093	-.001^a^	-.031	-.109^a^	.045	.135
Noise	-.061^a^	.012^a^	.107^a^	.000^a^	-.030^a^	-.001^a^	.072^a^
***SDT Measures*:**							
Perceptual Sensitivity (d’)	-.216	.052	.109^a^	.095	-.138^a^	.121	.263^b^
Response Bias (β)	.045	.087	-.087^a^	-.073	-.144^a^	-.007	-.026
***Paranormal Experiences*:**							
SAE No Responses	-	-.349^c^	-.583^a^^d^	-	-	-	-
Anomalous Responses	-	-	-.427^a^^d^	-	-	-	-
***Sensory-Processing Sensitivity*:**							
HSPS Score	-.301^b^	.058	.298^a^^b^	-	-	-	-
EOE	-.107^a^	.048^a^	.108^a^	.824^a^^d^	-	-	-
LST	-.169	.114	.158^a^	.837^d^	.603^a^^d^	-	-
AES	-.439^c^	.114	.279^a^^b^	.733^d^	.445^a^^d^	.452^d^	-

All DFs = 61.

^a^Spearman’s Rho correlations conducted due to non-normality.

^b^*p* < .05

^c^*p* < .01

^d^*p* < .001

To test the paradox between SPS, paranormal experiences, and perception, a multiple regression was calculated to predict Paranormal Responses based on two perceptual measures (yes responses in the EVP (pareidolia) condition and degraded human speech condition) as well as HSPS score and its three subscales. A significant regression equation was found, *F*(6,54) = 4.594, *p* < .001. The model explained 33.8% of the variance, and all variables significantly contributed to the model (*p* < .01), except for the percentage of yes responses in the EVP and degraded speech conditions (*p* > .05).

**Fig 2:**
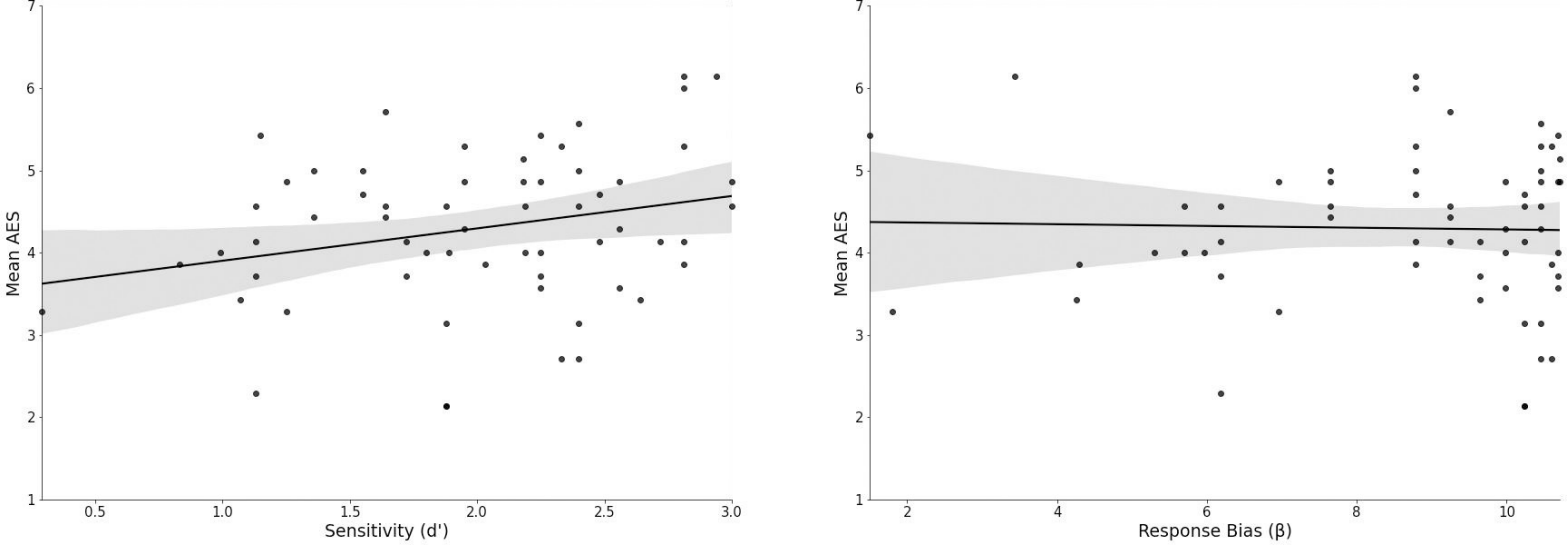
The relationship of AES with Signal Detection Theory measures of perceptual sensitivity and response bias. Scatterplots with confidence intervals demonstrating the relationship between AES and sensitivity (d’) and the lack of association between AES and response bias (β). Larger d’ values indicate greater tendency to differentiate signals from noise whereas larger β values correspond to a bias towards responding “No” (no voices present) [47].

To further explore the relationship with AES and perception, SDT measures were calculated to test sensitivity (d’) to perceiving voices where voices were present (hits, detection of degraded human speech) versus when they were not (false alarms, detection of voices in the noise condition), as well as response bias (β) (i.e., the inclination for participants to respond with more “Yes” responses or “No” responses). AES was significantly associated with perceptual sensitivity (d’), *r*(61) = .263, *p* = .04, but not to response bias (β), *r*(61) = -.026, *p* = .842 (Fig 2). Correlations between SDT measures and the remaining subscales and SAE Responses were non-significant (*p* > .05) (Table 1), and there were no significant differences between primed and unprimed participants for perceptual sensitivity (*t*(61) = 0.23, *p* = .82) or response bias (*t*(61) = -0.36, *p* = .72).

Finally, participants’ guesses were extracted for each trial (34 trials) within the degraded speech condition, and the number of correct guesses per participant was calculated. Overall, the number of correct guesses was low across the sample, ranging from 0 to 6, with a mean of 1.43 (SD = 1.50). This suggests that accurate speech recognition was difficult for participants. Due to the non-normality of the number of correctly identified degraded words, a Spearman’s rho was conducted and revealed a non-significant correlation between AES and the number of correctly identified degraded words (*r*_*s*_(61) = .07, *p* = .61), as well as with HSPS score (*r*_*s*_(61) = .11, *p* = .39).

**Fig 3:**
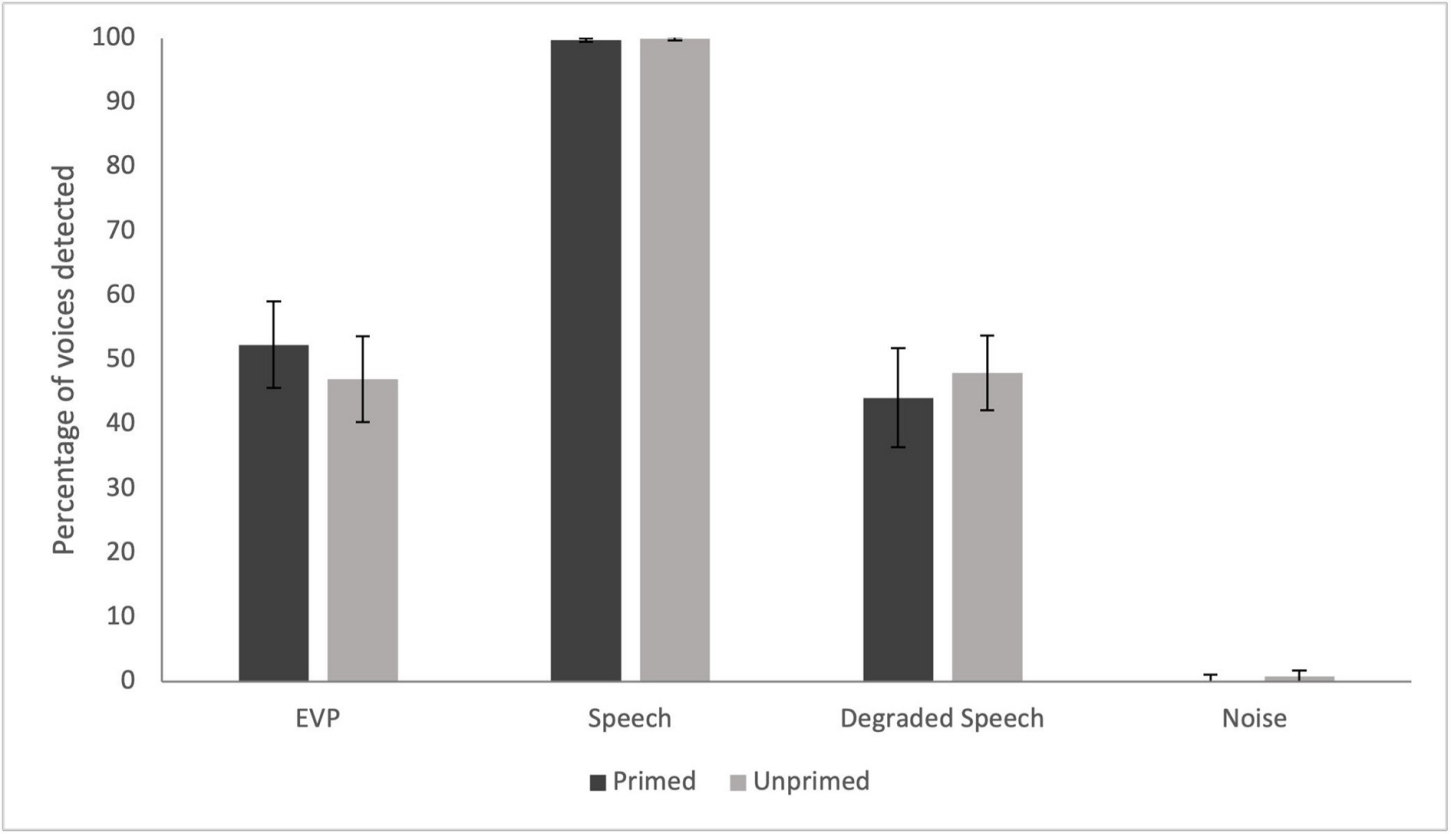
The percentage of words heard in each stimulus condition for the primed and unprimed groups. The mean percentage of yes responses in the EVP, speech, degraded speech, and noise stimulus conditions. Error bars represent 95% confidence intervals.

Replicating the analysis of Nees and Phillips [14], a 2 (instructional priming group) x 4 (stimulus condition) mixed ANOVA was conducted with percentage of yes responses as the dependent variable: due to the design of the current study, Paranormal Responses and SAE No Responses were added as covariates. The assumption of sphericity was violated, and thus Greenhouse-Geisser correction was used. A significant main effect of stimulus condition on yes responses was revealed, *F*(1.78, 101.52) = 29.42, *p* < .001, η_p_^2^ = 0.34. Pairwise comparisons, with an adjusted significance threshold using Bonferroni correction (a = .008), were used to test the differences between the four speech conditions. There were significant differences between all four conditions of stimulus (*p* < .001), except between the degraded speech and EVP conditions (*p* > .05) (Fig 3, for 95% Confidence Intervals see S3 Table). On the other hand, there was a non-significant main effect of priming group, *F(*1, 57) = 0.04, *p* = .84, η_p_^2^ = .001. There were non-significant differences between the groups for the detection of voices in degraded speech (*t*(59) = -.72, *p* = .47, 95% CI [-14.686, 6.691]), EVP (*t*(59) = 1.13, *p* = .26, CI [-4.116, 14.799]), speech (*t*(46.64) = -1.05, *p* = .30, 95% CI [-0.580, 0.182]), and noise (*t*(33.28) = -1.10, *p* = .14, 95% CI [-1.873, 0.557]). There was also a non-significant interaction between group and stimulus type, *F*(1.78, 101.52) = 1.43, *p* = .24, η_p_^2^ = .025. The same results held without inclusion of the covariates (as conducted in Nees and Phillips [14]).

Nees and Phillips [14] noted the violations of the assumption of normality for both the noise and speech conditions (see footnote 2, p.29), which is seen to be the case in the current study. With these conditions omitted, there was no change to the original authors’ findings of two significant main effects and a significant interaction. However, removing these and conducting a 2 (group) x 2 (stimulus condition) ANOVA with the current data (with Paranormal Responses and SAE No Responses as covariates) reveals a significant interaction between group and stimulus condition, *F*(1,57) = 4.08, *p* = .048, η_p_^2^ = 0.07), although the main effects were non-significant: stimulus condition, *F*(1,57) = 0.16, *p* = .69; group, *F*(1,57) = 0.09, *p* = .77. For the primed group, a paired samples t-test revealed a significant difference between these two stimulus conditions, *t*(29) = -2.47, *p* = .02, 95% CI [-15.057, -1.414]. The primed participants reported hearing a higher percentage of voices in the EVP stimuli compared with the degraded speech stimuli. However, for the unprimed group, there was a non-significant difference between these conditions, *t*(30) = 0.38, *p* = .71, 95% CI [-5.080, 7.972]. This suggests that participants informed of the paranormal aspect of the study reported hearing more voices in the EVP than the degraded speech condition (Fig 3).

Qualitative data (participants’ word guesses) were collected and analysed for the agreement amongst participants as to the spoken content of the stimuli within the four conditions. A total of 3,954 responses included scoreable guesses as to what the voices within the recordings had said. Scoreable guesses did *not* include words such as ‘I don’t know’, ‘Not sure’, ‘Man’s voice’, or ‘Interference’, and following the previous researchers, partial matches or rhyming words were not counted as in agreement, but homophones (e.g., ‘here’, ‘hear’) and spelling mistakes were. There was no trial in the noise condition in which more than one participant agreed upon the spoken content, thus, with the three remaining stimulus conditions, each trial (stimulus) with more than one response was analysed and the maximum percentage agreement across participants was calculated. This was done by identifying the word with which the most agreed and dividing the number of participants who agreed with the word by the total number of guesses for that trial.

Analysing differences between the percentage agreements in each stimulus condition (EVP, speech, and degraded speech), a one-way ANOVA was conducted. Stimulus condition was the independent variable, with each trial as a case (102 cases in total), and the mean percentage agreement as the dependent variable. The ANOVA revealed a significant difference between the stimulus conditions, *F*(2, 99) = 391.16, *p* < .001, η_p_^2^ = .89. Bonferroni post-hoc comparisons were used to explore the differences between these, with an adjusted alpha value of a = .017. There were differences between the degraded speech and speech conditions (*p* < .001), and between speech and EVP (*p* < .001). However, there were no significant differences between the EVP and degraded speech conditions, the two types of ambiguous stimuli (*p* > .05). Descriptive statistics are displayed in Table 2.

**Table 2:** Descriptive statistics for the maximum agreement of the spoken word content per condition.

	Mean	SD	Minimum	Maximum
Human Speech	89.03	17.78	31.15	100.00
Degraded Speech	10.69	10.31	0	40.00
EVP	11.80	10.17	0	43.33

The means, standard deviations (SD), minimum, and maximum values for the maximum spoken content agreement in each stimulus condition. Noise was not included as there was no trial on which more than one participant guessed the same word.

Lastly, participants’ guesses of the spoken content of the EVP stimuli were compared with the interpretations of the original paranormal researchers (Ghost Adventures). The original videos in which the EVP were extracted were subtitled with interpretations of the spoken content. As above, homophones and spelling mistakes were scored as being in agreement, but partial matches were not. 34 trials of EVP were included, with 984 scorable guesses: only 9 responses (0.91%) agreed with the paranormal researchers’ interpretations.

## Discussion

With large numbers of paranormal experiences reported in the general population [3], understanding these and their impact on thoughts, behaviour, and experiences is essential. The current study aimed to replicate the findings of Nees and Phillips [14], as well as extend this to test the associations of detection of ambiguous stimuli, priming, and paranormal experiences with sensory-processing sensitivity (SPS). The original findings were partially replicated, and support was found for the associations between paranormal experiences and SPS and SPS and accurate perception.

Firstly, there were no associations found between the reporting of paranormal experiences and perception, revealing no indication of a susceptibility to pareidolia in those who have had previous paranormal experiences. This contradicts the previous research which suggests that paranormal believers and potentially experiencers are more susceptible to misperceiving patterns in ambiguity [6, 7]. Furthermore, no support was found for experiencers of the paranormal being more susceptible to priming, although previous research has demonstrated that believers and experiencers are more likely to interpret anomalous phenomenon as paranormal, specifically when this is suggested to them [9, 11, 50].

Two explanations are provided for the lack of findings. First, it is possible that the perception of voices within EVP (pareidolia) is not related to paranormal experiences as measured by the SAE. Thus, alternative measures of paranormal experiences may provide different results. The second possible explanation concerns the definition of EVP as pareidolia, in that presenting experimentally compiled EVP to measure the tendency to perceive patternicity may be erroneous. The initial perception of voices within white noise can be defined as pareidolia, however, spontaneous pattern perception is eliminated once sounds (voices) have been identified and re-presented, the original perceiver having already attributed these signals as meaningful. In this case, the original perceiver of the EVP stimuli may have experienced pareidolia upon listening. Presenting to others as meaningful and voice-like encourages the perception of voices in these noises, and thus eradicates the randomness and spontaneity of the original pattern perception. Importantly, this poses implications on the use of these stimuli to objectively measure illusory perception. Future research should aim to investigate auditory pareidolia with random signals or noise that have not been attributed as meaningful prior to the experiment, enabling the ability to test spontaneous pattern perception. One suggestion, with the adequate resources, is allowing participants to record their own EVP sessions [16] and identify patterns within their recordings. However, it would be important here to differentiate misperception due to a natural tendency to identify patterns in randomness and misperception that is enhanced due to a priming effect, i.e., the suggestion that participants will capture meaningful recordings [13].

Secondly, the number of Paranormal Responses provided was significantly related to HSPS Score, suggesting that highly sensitives report a larger frequency of prior paranormal experiences. This is supportive of previous findings by Irwin et al [40] and Williams et al [41] and extends the research focusing on alternative types of sensitivity [34, 37–39]. A potential mediator of the relationship between SPS and paranormal experiences could be openness to experience, a trait that has been previously associated with both SPS [18, 32] and reporting paranormal experiences [51]. Open individuals tend to report a higher number of these incidents due to their inherent flexibility and experience-seeking behaviours [51], and thus, if HSPs are more open, this may leave them vulnerable to finding themselves in situations whereby typical, perceptual experiences can be interpreted as paranormal. This is simply speculation, and future research should fully explore this mediation effect further.

Although there was a lack of support for HSPS score being related to the detection of true degraded stimuli, it was found that a higher score on the subscale AES was related to a higher proportion of voices detected within the degraded human speech condition. This was also demonstrated using SDT measures, which distinguish perceptual sensitivity from response bias, in that AES scores were associated with heightened ability to distinguish signals from noise and not with the tendency to respond with bias. These findings partially support the perceptual advantage of SPS and the multi-dimensionality of the HSPS, such that AES might represent an aspect of sensitivity that differs from the other subscales, EOE and LST [32].

AES is defined as an awareness of aesthetic subtleties, leading to more positive emotional, sensory, and perceptual experiences [29, 30]. AES also has associations with positive elements of personality and behaviours, such as openness to experience and helping behaviours in the workplace [31, 32]. Therefore, these findings suggest that it is the AES aspect of SPS that captures the depth of processing described by previous research [18, 19, 27, 28, 41]. This finding can prove useful in highlighting that high SPS is not a deficit and can result in beneficial behaviours, depending on the environment. Demonstrating such advantages can aid in the understanding of SPS, such as in therapeutic environments, which support and promote the flourishing of HSPs in their everyday lives [19].

Furthermore, there were no associations between SPS (or the subscales) and the number of correctly identified words in the degraded speech condition. This could imply that SPS (AES) may be associated with stimulus fluency (detecting presence of voices) but not accuracy in identifying the content of such stimuli (spoken words). Nevertheless, the number of correctly identified words was very low across the entire sample (ranging from zero to six), suggesting that the signal to noise ratio simply created too much ambiguity and degradation to allow for accurate speech identification, regardless of SPS. Such a high level of degradation was the aim of the original researchers [14] upon creation of the stimuli. Thus, it would be interesting to explore degradation levels at which accurate stimulus detection and identification can occur, and the associations of this with SPS, so as to test thresholds for accurate stimulus detection and identification.

Finally, Nees and Phillips [14] demonstrated that a simple instructional prime resulted in a perceptual shift; participants that were told it was a study containing paranormal recordings heard more voices within the ambiguous stimulus conditions. Contrary to this, and with a larger sample of participants, the prime in the current study did not introduce differences between the primed and unprimed participants in this measure, both groups identified a similar proportion of voices across the stimulus types. However, an alternative priming effect was revealed: primed participants detected significantly more voices within the EVP condition compared with the degraded human speech, whereas this difference did not occur for the unprimed group. These findings imply that the EVP stimuli produced a unique response in those expecting to hear “ghost voices”, perhaps due to the spooky, ominous qualities that these EVP recordings possess compared with the artificially produced degraded speech. Where there is no existing expectation of hearing ghostly voices, both types of ambiguous recordings are interpreted similarly. This emphasises the importance of prior expectations on paranormal perception, as well as the notion that expectations could lead to false conclusions in real-world situations [12, 52, 53].

As found previously, within primed and unprimed groups there was a lack of agreement between participants on the spoken content of the EVP and degraded speech stimuli. Furthermore, there was little to no agreement on the apparent spoken content of EVP items between the participants in the current study and the original Ghost Adventures researchers’ interpretations of the voices. Thus, although there seems to be acoustical differences in the way pareidolic and truly degraded stimuli are perceived, there are contextual similarities in individuals’ attempts to identify the spoken content of such recordings, evidencing the difficulty of non-contextual speech perception, and suggesting a role of top-down processes for ‘filling the gaps’ where sensory information is limited [15, 16].

### Limitations

Although the current study aimed to capture a more representative sample of participants than in Nees and Phillips [14], the majority (90%) of the participants were White and resided in Wales, United Kingdom. With notable differences between ethnicities in paranormal experiencing [54], the generalisability is questionable. Another important limitation to note is the sample size, which was sensitive enough only to detect an effect size of η_p_^2^ = .12 for the between-subjects effect of priming group. Thus, if the true effect size was smaller than this, the difference between groups would not be detectable.

In addition, the instructional prime was very subtle (only one sentence) and could have been easily missed by participants. If this occurred, then the prime was ineffective from the beginning and could explain the lack of difference between the perceptions of primed and unprimed participants. This is supported by the finding that only seven (24%) primed participants “guessed” the true nature of the experiment when asked at the end, the true nature being that there was a paranormal aspect to the investigation. However, it is plausible that participants misunderstood what this question was truly asking, for example, they may have thought the question was directed towards the perceptual and personality testing, for which they would have also been naïve from the outset. Thus, a greater prime may have elicited a stronger effect on participants’ perceptions. On the other hand, a subtle priming effect was found for the primed group only, such that they detected more EVP voices than degraded speech. Perhaps participants had unconsciously processed the paranormal aspects of the instructions which resulted in distorted perceptions of voices within random noise.

As mentioned previously, there was also a confound introduced by the nature of the stimuli used in the current paradigm. Specifically, the degraded human speech recordings were the same as those used in the human speech condition. This means that participants may have had a detection and identification advantage on trials where the human speech equivalent was heard prior to the degraded speech recording, leading to inflated scores in the degraded speech condition only. This has implications for the comparisons conducted with the other conditions, including the EVP condition (another type of ambiguous stimuli). Also, this “prime” may not have been consistent across participants in the sample, and specific individual differences (e.g., being a HSP) could determine variation in the effect of the confound across the sample. Therefore, generalisability issues arise, as the perceptual bias of presenting the human speech prior to the degraded speech may affect some individuals more than others. Furthermore, this confound could only be analysed for n = 40 participants due to a file error, which meant the original (trial) presentation order of n = 21 participants was lost and could not be restored. Future research adopting this paradigm should take this confound into consideration when determining the presentation order of stimulus conditions, perhaps fully testing the impact it has on perceptual ability and individual differences. Alternatively, the confound could be avoided by using different words for the speech and degraded speech conditions.

### Conclusion

Perceptual illusions and prior experience and belief can influence humans to erroneously misinterpret stimuli and situations, posing real world implications, such as errors in decision-making, pseudoscientific beliefs, and susceptibility to misinformation about global events [55, 56]. From the current findings, it is shown that increased reporting of paranormal experiences is not associated with the detection of voices within examples of EVP, and these individuals are no more susceptible to priming effects. Also, SPS and paranormal experiences are positively correlated, and the subscale of AES is related to increased perceptual sensitivity to detecting truly degraded stimuli, supported by SDT. We also conclude that the original priming effect found in Nees and Phillips [14] does not fully replicate, however, a simple instructional prime resulted in the detection of a larger proportion of ghost voices than of true, degraded human voices.

## Supporting information

S1 Fig Density distributions for the subscales of the HSPS. (TIF)

S1 Table Correlations between perception and paranormal experiences for the primed group only. The Pearson’s (r) and Spearman’s Rho (r_s_) correlations between the percentage of yes responses in each stimulus condition and SAE responses for primed participants.
(PDF)

S2 Table Correlations between perception and paranormal experienced for the unprimed group only. The Pearson’s (r) and Spearman’s Rho (r_s_) correlations between the percentage of yes responses in each stimulus condition and SAE responses for unprimed participants.
(PDF)

S3 Table Mean (M) differences between the stimulus conditions and 95% Confidence Intervals (CI [Upper, Lower]). (PDF)
