# Supplementary figures and images for: Paranormal experiences, sensory-processing sensitivity, and the priming of pareidolia
Source: PLoS One. 2022 Sep 14;17(9):e0274595. doi: 10.1371/journal.pone.0274595 (PMC9473424; doi:10.1371/journal.pone.0274595)

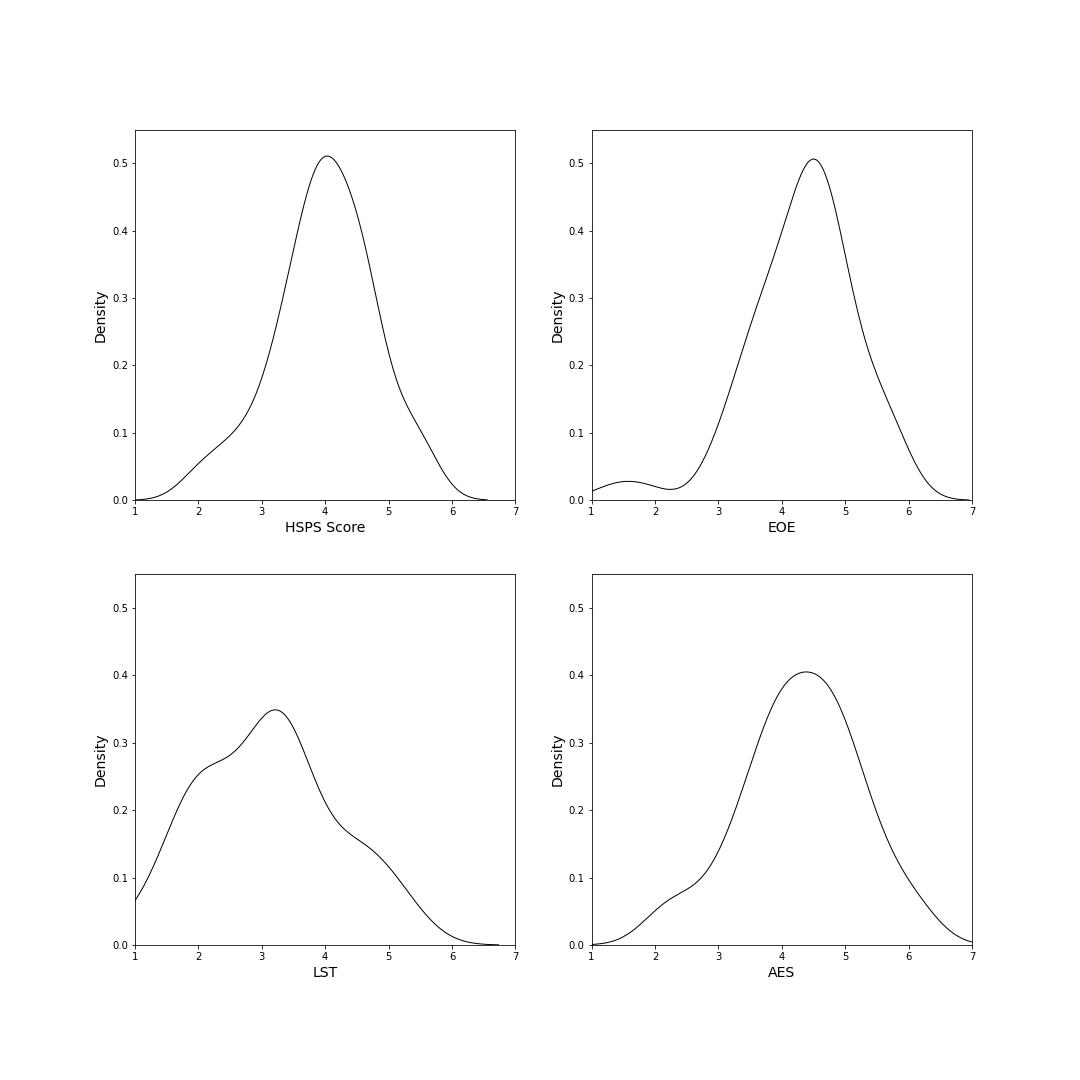

Supplement: S1 Fig — (TIF) [file pone.0274595.s001.tif]
