# Supplementary material for: Paranormal experiences, sensory-processing sensitivity, and the priming of pareidolia
Source: PLoS One. 2022 Sep 14;17(9):e0274595. doi: 10.1371/journal.pone.0274595 (PMC9473424; doi:10.1371/journal.pone.0274595)
Supplement: S1 Table — The Pearson’s (r) and Spearman’s Rho (rs) correlations between the percentage of yes responses in each stimulus condition and SAE responses for primed participants. (PDF) [file pone.0274595.s002.pdf]

S1 Table. Correlations between perception and paranormal experiences for the primed group only. The Pearson's (r) and Spearman's Rho (r_s) correlations between the percentage of yes responses in each stimulus condition and SAE responses for primed participants.

Primed	SAE No Responses	Anomalous Responses	Paranormal Responses
Degraded Speech	r = -.279, p = .135	r = .001, p = .994	r_s = .294, p = .114
Human Speech	r_s = .193, p = .307	r_s = .061, p = .748	r_s = -.144. p = .448
EVP	r = -.167, p = .377	r = .197, p = .298	r_s = -.122, p = .521
Noise	r_s = .052, p = .783	r_s = .075, p = .693	r_s = -.064, p = .738
