# Supplementary material for: Paranormal experiences, sensory-processing sensitivity, and the priming of pareidolia
Source: PLoS One. 2022 Sep 14;17(9):e0274595. doi: 10.1371/journal.pone.0274595 (PMC9473424; doi:10.1371/journal.pone.0274595)
Supplement: S2 Table — The Pearson’s (r) and Spearman’s Rho (rs) correlations between the percentage of yes responses in each stimulus condition and SAE responses for unprimed participants. (PDF) [file pone.0274595.s003.pdf]

S2 Table. Correlations between perception and paranormal experienced for the unprimed group only. The Pearson's (r) and Spearman's Rho (r_s) correlations between the percentage of yes responses in each stimulus condition and SAE responses for unprimed participants.

Unprimed	SAE No Responses	Anomalous Responses	Paranormal Responses
Degraded Speech	r = -.134, p = .474	r = .103, p = .582	r = -.033, p = .861
Human Speech	r_s = -.288, p = .116	r_s = .248, p = .179	r_s = .182, p = .326
EVP	r = -.112, p = .548	r = .017, p = 927	r = .051, p = .784
Noise	r_s = -.124, p = .505	r_s = -.032, p = .863	r_s = .251, p = .173
