# Supplementary material for: Paranormal experiences, sensory-processing sensitivity, and the priming of pareidolia
Source: PLoS One. 2022 Sep 14;17(9):e0274595. doi: 10.1371/journal.pone.0274595 (PMC9473424; doi:10.1371/journal.pone.0274595)
Supplement: S3 Table — (PDF) [file pone.0274595.s004.pdf]

S3 Table. Mean (M) differences between the stimulus conditions and 95% Confidence Intervals (CI [Upper, Lower]).

	Degraded Speech		EVP		Speech		Noise	
	**M Difference**	**CI [U,L]**	**M Difference**	**CI [U,L]**	**M Difference**	**CI [U,L]**	**M Difference**	**CI [U,L]**
**Degraded Speech**	-	-	3.619	-8.117, 0.880	53.737	-59.157, -48.318	-45.543	40.198, 50.888
**EVP**	-3.619	-0.880, 8.117	-	-	50.119	-54.918, -45.320	-49.162	44.444, 53.879
**Speech**	-53.737	48.318, 59.157	-50.119	45.320, 54.918	-	-	-99.280	98.627, 99.934
**Noise**	45.543	-50.888, -40.198	49.162	-53.879, -44.444	99.280	-99.934, -98.627	-	-
